# Emerging links between m^6^A and misregulated mRNA methylation in cancer

**DOI:** 10.1186/s13073-016-0395-8

**Published:** 2017-01-12

**Authors:** Samie R. Jaffrey, Michael G. Kharas

**Affiliations:** 1 Department of Pharmacology, Weill Cornell Medical College, Cornell University, New York, NY 10065 USA; 2 Molecular Pharmacology Program, Memorial Sloan Kettering Cancer Center, New York, NY USA

## Abstract

*N* ^6^-methyladenosine (m^6^A) in mRNA has emerged as a crucial epitranscriptomic modification that controls cellular differentiation and pluripotency. Recent studies are pointing to a role for the RNA methylation program in cancer self-renewal and cell fate, making this a new and promising therapeutic avenue for investigation.

## m^6^A, an epitranscriptomic mark that influences cellular differentiation

One of the hallmark features of cancer is misregulated gene expression. A newly recognized concept in the regulation of gene expression is that mRNAs contain a diverse set of modified nucleotides, and the location and identity of these modifications within the transcriptome constitute an ‘epitranscriptomic’ code. The initial concept of the epitranscriptome was introduced as a result of transcriptome-wide mapping of *N* ^6^-methyladenosine (m^6^A), which revealed that m^6^A is found in at least a quarter of all mRNAs, typically near stop codons [1].

RNA methylation is mediated by a multiprotein ‘writer’ complex comprising RBM15–WTAP–METTL3–METTL14 [2]. METTL3 is the sole methyltransferase responsible for forming m^6^A, whereas RBM15 couples the methylation complex to mRNA to methylate adjacent m^6^A residues [2]. WTAP acts as an adaptor, coupling RBM15 to METTL3, whereas METTL14 positions RNA substrates for methylation by METTL3. Notably, adenosine methylation is reversible. Although FTO (fat mass and obesity-associated protein) was reported to be an m6A demethylase, it is now known that AlkB family member 5 (ALKBH5) is the only enzyme to show physiologically relevant demethylation activity in vivo [3].

Under normal conditions, the most prominent effect of the presence of m^6^A is to induce mRNA degradation, but, in response to certain types of cellular stress, the m^6^A distribution across the transcriptome can change, with the most notable effect being increases in the abundance of m^6^A marks in the 5′-untranslated region of select mRNAs [4]. This methylation confers to the mRNA the ability to be translated in a manner that does not require the canonical cap-binding protein eIF4E [4]. eIF4E-independent translation is activated in diverse disease states, especially cancer.

**Fig. 1:**
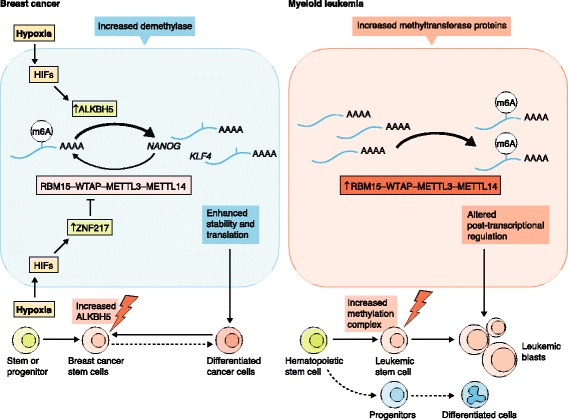
Cancer can be promoted by upregulating either *N* ^6^-methyladenosine (*m* ^*6*^ *A*) demethylases or methyltransferase proteins. In breast cancer, hypoxia increases the expression of ALKBH5 or ZNF217 through the activation of hypoxia-inducible factors (*HIFs*). ALKBH5 is an m6A demethylating enzyme, and ZNF217 inhibits the RNA methylation writer complex (RBM15–WTAP–METTL3–METTL14), resulting in a reduction of the levels of the m^6^A modification in the mRNA of breast cancer pluripotency transcripts *NANOG* and *KLF4*, promoting their stability and increased expression. This contributes to the reacquisition of the breast cancer stem cell phenotype in these cells. In myeloid leukemia, by contrast, increased levels of components of the m^6^A methylation machinery proteins (RBM15–WTAP–METTL3–METTL14) are present, suggesting misregulated and increased mRNA methylation. Thus, the increase in these proteins might alter the normal differentiation trajectory of hematopoietic stem cells, leading to abnormal fates, including leukemic blasts. (*Arrows* indicate activation; ‘*lightning bolts*’ indicate misregulation of the RNA methylation program)

A connection between m^6^A and cancer-relevant processes is suggested from studies linking m^6^A to differentiation pathways that control stem cell fate [5]. Pluripotent stem cells depleted of m^6^A show marked resistance to stimuli that promote differentiation. These cells retain pluripotency markers and fail to acquire gene expression patterns seen in differentiated cells. By contrast, primed stem cells, which lack the ability to contribute to blastocyst chimeras, are more prone to differentiate and show enhanced and abnormal expression of differentiation markers upon depletion of m^6^A [5]. These studies show that alterations in m^6^A levels can alter differentiation pathways. As the pathways involved in embryonic stem cell maintenance and differentiation have been directly linked to the acquisition of stem cell properties in both solid and hematological malignancies, m^6^A alterations might have a role in cancer development (Fig. 1). Hypoxic environments and dysregulation of hypoxia-inducible factors (HIFs) have been implicated in a variety of cancers, including brain, lung, pancreatic, colon, ovarian, and many other cancers.

## ALKBH5 and m^6^A depletion as a driver of cancer stem cell formation

In line with the above, recent studies point to a link between alterations in m^6^A levels and the abnormal cellular differentiation states present in cancer. In a variety of tumors, cancer stem cell populations are readily detected in hypoxic niches. Semenza and colleagues showed that hypoxia was associated with increased breast cancer stem cell formation and elevated levels of ALKBH5 in breast cancer [6]. Notably, ALKBH5 was required for acquisition of the breast cancer stem cell phenotype. The authors observed that hypoxia increased the stability of *NANOG* mRNA and its protein levels. This could reflect ALKBH5-mediated *NANOG* mRNA demethylation or an indirect effect of ALKBH5 expression on m^6^A levels in *NANOG* mRNA [6]. NANOG is a key transcription factor that is associated with pluripotency. In addition to the effects of hypoxia on *NANOG* mRNA, hypoxia induces the expression of the zinc-finger protein ZNF217, which inhibits nuclear methylation [6]. Induction of ZNF217 also reduces m^6^A levels in *NANOG*, as well as in *KLF4* [7]. The KLF4 transcription factor is a pluripotency factor required for the maintenance of breast cancer stem cells. Thus, hypoxia reduces m^6^A levels to promote the formation of breast cancer cells (Fig. 1).

## m^6^A elevations in hematologic malignancies

While hypoxia decreases m^6^A levels, increases in the abundance of m^6^A might also predispose to cancer. This is supported by evidence that genes that encode proteins that contribute to the RNA methylation complex are upregulated in myeloid leukemia (Fig. 1). Analysis of The Cancer Genome Atlas (TCGA; https://cancergenome.nih.gov/) shows that METTL3, METTL14, and RBM15 are highly expressed in myeloid leukemia compared with other cancers. These proteins appear to be required for maintaining the abnormal differentiation state seen in myeloid leukemia. A role for m^6^A in myeloid leukemias is supported by studies of WTAP depletion. Bansal and colleagues found that WTAP expression was elevated in cells derived from 32% of patients with acute myeloid leukemia [8]. WTAP knockdown results in reduced proliferation, increased differentiation, and increased apoptosis in a leukemia cell line [8]. WTAP knockdown is a highly efficient approach to deplete m^6^A from mRNA. Thus, m^6^A depletion might account for the anti-leukemia effects observed upon WTAP depletion.

RBM15, another component of the m^6^A writer complex, is also linked to myeloid leukemia. In this case, RBM15 has a clear driver role in the development of hematologic malignancy. Acute megakaryoblastic leukemias were shown to be mediated by a chromosomal translocation t(1;22) of *RBM15* (also called *OTT1*) with the *MAL* gene [9]. RBM15 has crucial roles in maintaining quiescence in hematopoietic stem cells and in megakaryocyte leukemia cell line differentiation by controlling the splicing of key differentiation genes, including *GATA1*, *RUNX1*, *TAL1*, and *c-MPL* [10]. Because RBM15 directs m^6^A formation in the transcriptome [2], the oncogenic effects of RBM15 overexpression and *RBM15-MAL* translocation might reflect aberrant m^6^A formation.

Although each of the major proteins in the m^6^A methylation complex—that is, RBM15, WTAP, METTL3, and METTL14—show alterations in myeloid leukemias, definitive demonstration of the role of m^6^A will require mechanistic evidence linking m^6^A alterations to leukemia phenotypes in these cancers.

## Conclusions

The modification m^6^A is an epitranscriptomic mark that influences a wide variety of RNA processing steps, including splicing, mRNA stability, and translation. Genes associated with pluripotency and lineage-specific differentiation are controlled by m^6^A levels, and reduced m^6^A levels can lead to a misregulation of these genes and the acquisition of stem cell characteristics. Alternatively, increases in m^6^A levels are expected to stabilize these transcripts and would therefore be particularly problematic in tissues that are continuously replenished from a stem cell population, such as the hematopoietic lineage. Hematopoietic stem cells traverse through distinct differentiation intermediates in order to achieve their final differentiated state. Elevations in m^6^A might alter the normal differentiation pathway, resulting in cells being trapped in a progenitor cell state.

Many unanswered questions remain. How conserved are these pathways in other cancer types? Many cancer subtypes are associated with abnormal differentiation states or cancer stem cells, making it likely that interventions that influence m^6^A levels could therapeutically alter the differentiation program. Will a systematic analysis of the marked transcripts in cancer reveal new targets for therapeutic intervention? Can pharmacologic modulation of the RNA methylation program in various cancers push cells toward differentiation? Another important question is whether targeting m^6^A would have unwanted side-effects. As m^6^A might be used in every cell for the regulation of gene expression, targeting m^6^A might not provide a suitable therapeutic index. Finally, the high reliance of myeloid leukemia cells on methylation complex proteins raises the hope that these cells will show higher sensitivity to m^6^A pathway inhibitors than other cell types.

## Abbreviations

m^6^A: *N* ^6^-methyladenosine
TCGA: The Cancer Genome Atlas
